# Effects of Vestibular Rehabilitation on Physical Activity and Subjective Dizziness in Patients With Chronic Peripheral Vestibular Disorders: A Six-Month Randomized Trial

**DOI:** 10.3389/fneur.2021.656157

**Published:** 2021-04-29

**Authors:** Tomoyuki Shiozaki, Taeko Ito, Yoshiro Wada, Toshiaki Yamanaka, Tadashi Kitahara

**Affiliations:** Department of Otolaryngology-Head and Neck Surgery, Nara Medical University, Kashihara, Japan

**Keywords:** vestibular rehabilitation, physical therapist, physical activity, randomized control trail, chronic peripheral vestibular disorders

## Abstract

**Introduction:** The present study aimed to determine whether supervised vestibular rehabilitation therapy (VRT) by physical therapists (PTs) affects subjective dizziness in patients with chronic vestibular disorders, and whether supervised VRT-induced changes in subjective dizziness are related to the changes in physical activity levels in daily life.

**Methods:** Patients (*n* = 47) with chronic peripheral vestibular disorders were randomly divided into the VRT group (*n* = 25) and control group (*n* = 22). Patients in the VRT group received weekly supervised visits from PTs for a period of 6 months. Every other month, both groups were advised by neuro-otologists to increase the amount of activity in their daily life. All patients wore an accelerometer device, which recorded their physical activity for seven successive days before the end of the intervention. Patients also completed the dizziness and unsteadiness questionnaires before and after the intervention.

**Results:** Subjective dizziness decreased significantly regardless of whether supervised VRT was administered; however, dizziness evoked by social activity and head and body movements improved more significantly in the VRT group than in the control group. In the VRT group, there was a significant negative correlation between the increase in sedentary behavior and improvement in subjective dizziness, and a significant positive correlation between the increase in light physical activity and improvement in subjective dizziness at the second month of intervention. The VRT group showed a significantly higher rate of increase in light physical activity than the control group, after 6 months of intervention.

**Conclusion:** Supervised VRT could be highly effective in treating subjective dizziness in patients with chronic peripheral vestibular disorders. We believe frequent (weekly) and medium-term (6 months) PT-guided interventions may be highly effective in enhancing physical activity in daily life, and may subsequently improve subjective dizziness in these patients.

**Trial registration:** This clinical study was registered with University hospital Medical Information Network (identification number: 000028832). https://www.umin.ac.jp/

## Introduction

Individuals with dysfunction of the vestibular system report symptoms such as, disorientation, lightheadedness, disequilibrium, and visual blurring (1). These symptoms decrease with an increase in vestibular compensation. However, a slow rate of vestibular compensation and prolonged dizziness are often observed (2).

A vicious cycle of dizziness is thought to be one of the causes for vestibular decompensation-induced chronic dizziness (3). Symptoms of dizziness act as the starting points, as these symptoms initially cause anxiety and fear, leading to avoidance of activities. Avoidance of activities that might provoke dizziness not only causes delayed vestibular compensation, but also leads to depression and restricted social behavior, which reduces the quality of life in the long term. A previous study, using a wearable device to monitor physical activity, showed that in patients with dizziness, sedentary behavior (SB) was higher and light-intensity physical activity (LPA) and total physical activity were lower than in healthy adults (4). In a previous study, more than 25% of patients with dizziness reported difficulty in performing activities associated with 29 of the 34 items listed in the activity questionnaire (5). In patients who are in a vicious cycle of dizziness, it is important to present the diagnostic conclusions positively once the medical investigation has been completed. Patients should be educated to increase their physical activity and promote vestibular compensation (3).

One of the treatments for patients with vestibular decompensation is vestibular rehabilitation therapy (VRT), which aims to promote vestibular compensation by focusing on exercises that improve postural stability and gaze functions associated with head movements, thereby decreasing restrictions of activities of daily living, and enhancing the quality of life by encouraging social participation. The types of vestibular rehabilitation have become remarkably varied over the years, ranging from group exercises and customized exercise programs to internet-based programs (6–8). In several randomized trials, VRT reportedly improved subjective dizziness, postural stability, gait speed, and dynamic visual acuity (9–13). If the delay in vestibular compensation is due to a vicious cycle of vertigo, then lifestyle guidance from neuro-otologists (N-Os) may be a sufficient treatment. However, it is unknown whether the effects of VRT are influenced by the special training performed under the supervision of the physical therapists (PTs), by the increased physical activity and vestibular compensation promoted by lifestyle guidance, or by both. It has also been suggested that it is important to increase the amount of physical activity that was reduced due to the vicious cycle of vertigo. However, there are no reports that objectively verify the relationship between increased activity and improvement in dizziness.

The objectives of the present randomized controlled study were: (1) to examine whether VRT under the supervision of PTs is more effective than lifestyle guidance in improving subjective dizziness, and (2) to investigate the relationship between VRT-induced changes in subjective dizziness and enhanced physical activity in their daily life.

## Methods

This clinical study was registered with UMIN (identification number: 000028832). The use of all patients' data pertaining to this study was approved by the Ethics Committee of the Nara Medical University Hospital (identification number: 0889). The patients provided written informed consent to participate in this study.

### Patients

Before conducting the trial, the appropriate sample size was estimated by power analysis using software G^*^power (Version 3.1.9.4) (14) for a two-way analysis of variance (ANOVA). The effect size f was set to 0.4, alpha error probability to 0.05, beta error probability to 0.90, number of groups to 2, and number of measurements to 4. The calculated sample size was 36; therefore, 45 patients were recruited with an expected dropout of 20%.

One hundred and five patients with intractable vertigo/dizziness who were hospitalized for undergoing several neuro-otologic examinations at the Vertigo/Dizziness Center of the Nara Medical University between October 2017 and September 2019, were enrolled. The examinations included the caloric test (C-test), video head impulse test (vHIT), and cervical vestibular evoked myogenic potentials (cVEMP).

These examinations were performed in accordance with previous reports (15–17). Briefly, the C-test was performed by injecting cold water (20°C; 20 mL) into the external auditory meatus over the course of 10 s, and the induced nystagmus was recorded using electronystagmography (ENG). Based on the maximum slow-phase eye velocity, the caloric test was classified as pathological when the ENG response was ≤10 deg/s. The vHIT was also used to assess the vestibulo-ocular reflex (VOR) in the three semicircular canals using a lightweight video-oculography device (ICS Impulse; GN Otometrics, Taastrup, Denmark). The normal VOR gains were set to ≥0.8 (horizontal canals) and ≥0.7 (vertical canals). The vHIT was considered pathological when the gain was lower than the reference value, and a catch-up saccade was observed. The cVEMP assessed the first biphasic response (p13–n23) produced by the sternocleidomastoid muscle ipsilateral to the acoustically stimulated ear. In the present study, a right-left or left-right ratio in activity below 0.5 was considered pathological. In this study, the absence of bilateral VEMP was not considered pathological, as bilateral VEMP may be absent in healthy elderly people (18).

Patients who met the following criteria were enrolled in the study: (1) unilateral or bilateral peripheral vestibular hypofunction with pathological results on at least one of the three tests, (2) age between 20 and 85 years, and (3) presence of symptoms of dizziness and imbalance for at least 6 months. Subjects were excluded if they showed: (1) involvement of the central nervous system, (2) spontaneously fluctuating and intermittent vertigo, and (3) significant orthopedic or cardiac problems.

**Figure 1:**
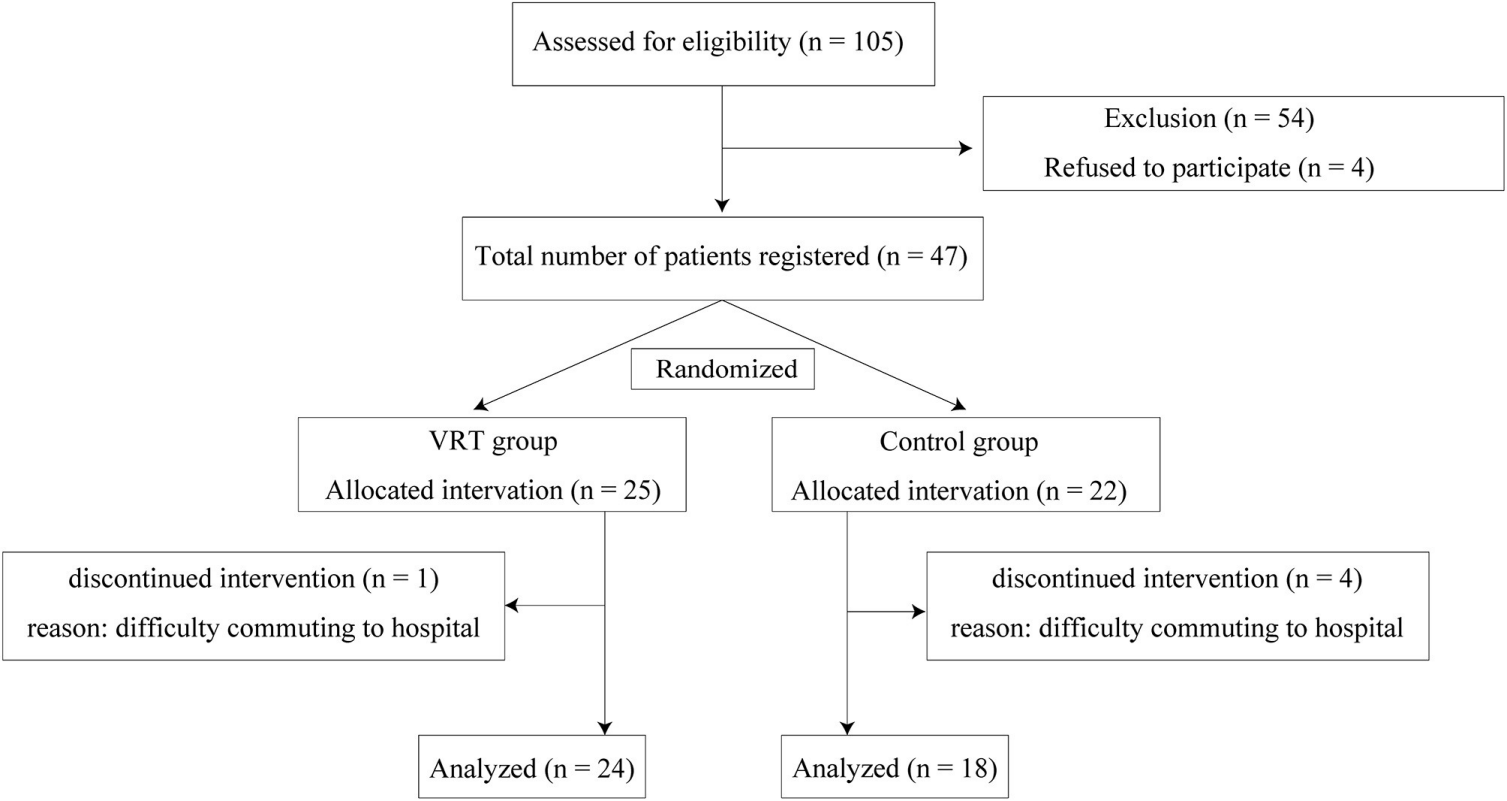
Flow diagram of the study.

Among the 105 patients initially enrolled, 54 were excluded based on the exclusion criteria and 4 declined to participate; a total of 47 patients finally participated in the study. In accordance with the Declaration of Helsinki, all patients provided written informed consent after receiving a detailed explanation of the impending procedures. All patients were randomly assigned to one of the two following treatment groups, using a computer-generated simple randomization organized by the clinical study section at the Vertigo/Dizziness Center of the Nara Medical University: the VRT group and control group. One patient in the VRT group and four patients in the control group were unable to complete the program because of difficulty in commuting to hospital, and were excluded from the analysis (Figure 1).

Patients in the VRT group attended weekly VRT sessions conducted by PTs for 6 months, with independent daily practice. Patients in the control group received explanations regarding the disease and instructions on increasing activity in their lives from N-Os and PTs, every 2 months for 6 months. All patients were assessed using “the dizziness and unsteadiness questionnaire' before and after the intervention. Physical activity was also measured at the beginning and at month 2 and month 6 of the intervention. These evaluations were performed by author who was blinded regarding the groups to which the patients belonged.

### Physical Activity Measurement

Physical activity was assessed using the Active style pro HJA-750C (Omron Healthcare, Kyoto, Japan). This wearable device estimates the metabolic equivalents (METs) every 10 s, based on combined accelerations measured by a built-in triaxial accelerometer, that has a minimum threshold of 3 mG and a sampling frequency of 32 Hz. The criterion-related validity of the METs estimated by this device has been previously confirmed with the Douglas bag method (19). Patients wore the device on their lumbar region, except during water activities, from the time they woke up till they went to bed. Intensity of any activity that was below the detection threshold of 0 for ≥20 min, was considered non-worn time; data were adopted if the wearer wore the device for more than 10 h/day.

All participants in the present study wore the device at the beginning of the intervention, month 2, and month 6 until 7 days of physical activity were successfully recorded (20). Motor behaviors during the waking hours were classified into two types: SB and physical activity. Both, LPA and moderate to vigorous-intensity physical activity (MVPA) were included in physical activity. In this study, SB was defined as “awake activity characterized by energy expenditure of 1.5 METs in a sitting or reclining position,” LPA as activity between 1.5 and 3.0 METs, and MVPA as all activities over 3.0 METs (21). The ratio of duration of SB, LPA, and MVPA to total wear-time was also calculated.

### Dizziness and Unsteadiness Questionnaire

Use of this questionnaire is the best method to analyze subjective dizziness handicap data, because it facilitates the use of validated clinical metrics such as, “Dizziness Handicap Inventory” of Jacobson and Newman (22). “The dizziness and unsteadiness questionnaire” used in this study was developed from “Dizziness Handicap Inventory” and has been available for the evaluation of everyday handicap due to dizziness in the Japanese population, since 1995. It includes 14 principal questions; an English translation has been presented in Table 1 (23). For the assessment, the answers to all the principal questions were scored on a scale of 1 to 5: severe handicap = 5, significant handicap = 4, moderate handicap = 3, slight handicap = 2, and no handicap = 1 due to the symptom. The final score for each factor was calculated by adding all the individual scores for each of the three principal questions. The principal questions 1, 5, and 9 belong to factor 1 = disturbance of social activity due to dizziness; 2, 6, and 10 belong to factor 2 = body motion precipitating dizziness (head and sight); 3, 7, and 11 belong to factor 3 = limitation of physical activity (body movement); 4, 8, and 12 belong to factor 4 = emotional disturbance due to dizziness; and 1, 12, and 13 belong to factor 5 = disturbance of interpersonal communications due to dizziness.

**Table 1:** The dizziness and unsteadiness questionnaire.

1) Do you refrain from going out or travel for works or amusement due to dizziness or unsteadiness? (a) always (b) frequently (c) sometimes (d) rarely (e) no (f) no idea
2) Do you hate walking in the dark places even though around your home due to dizziness or unsteadiness? (a) absolutely (b) significantly (c) moderately (d) slightly (e) no (f) no idea
3) Do you hate going downstairs due to dizziness or unsteadiness?
4) Do you feel annoying due to dizziness or unsteadiness?
5) Do you feel that you are not able to do your work either at home or at office due to dizziness or unsteadiness?
6) Is the degree of dizziness or unsteadiness strengthened when you suddenly move your head (e.g., at turning back)?
7) Do you hate walking through the narrow spaces (e.g., narrow sidewalk) due to dizziness or unsteadiness?
8) Do you feel that you have a handicap in your body and are inferior to other persons due to dizziness or unsteadiness?
9) Don't you concentrate on something due to dizziness or unsteadiness?
10) Do you think it too much trouble to read books or newspaper due to dizziness or unsteadiness? Or do you have some trouble in reading them?
11) Is the degree of dizziness or unsteadiness strengthened when you stand up from a chair?
12) Do you feel anxiety about yourself when you are in the presence of others due to dizziness or unsteadiness?
13) Do you refrain from meeting or going out with your family or friends due to dizziness or unsteadiness?
14) Do you have difficulties in your daily due to dizziness or unsteadiness?

### Exercise Program

Patients in the VRT group underwent a developed version of the Cawthorne–Cooksey VRT (24, 25), called MAHOROBA (i.e., an ancient Japanese word for peaceful and comfortable place) in our facility, under the supervision of PTs. MAHOROBA-style VRT consists of adaptation, habituation, balance, and gait training, with a gradual increase across three difficulty levels. Patients received weekly 1-h sessions from PTs and performed daily independent exercises at home using a booklet. Home exercises were monitored using a chart that was filled-in every day by the patients. Patients in both groups were evaluated by N-Os every 2 months to explain their current situation and provide guidance on their lives. The medication status varies from patient to patient; no medication changes were made during the study period to prevent any impact on the results.

### Data Analysis

The statistical package for social sciences, SPSS 25 (SPSS Inc., Chicago, USA) for Windows was used for statistical analysis. Normal distributions of all data were tested using the Shapiro–Wilk test. Baseline characteristics were compared using the non-paired t- or Mann–Whitney *U-tests* (numerical data: age and length of illness) and the Fisher's exact test (nominal data: sex and diagnosis).

In each group, the differences in the scores for each factor on the pre- and post-intervention questionnaires were evaluated using two-way repeated measures ANOVA with group and time factors; Bonferroni correction was performed as *post-hoc* analysis. It has been reported that the Likert scale scores can be analyzed as ordinal scale scores using ANOVA (26, 27). In the present study, we used two-way repeated measures ANOVA to analyze the scores of questionnaires on the Likert scale, if they showed normal distribution.

To investigate the relationship between the changes in activity and subjective dizziness, we examined the relationship between the changes in final scores for each of the five factors in the dizziness and unsteadiness questionnaire from before to after the intervention; in the VRT and control groups, the changes in SB, LPA, and MVPA from baseline to 2 months and from pre- to post-intervention, were evaluated using the Spearman's rank correlation coefficient.

We calculated the percentage increase in SB, LPA, and MVPA from before to 2 months after the intervention and from the pre-intervention timepoint to the 6-month post-intervention timepoint. Differences in the change in activity levels between the groups were compared using an unpaired *t-test*. The significance level was set at <5%.

## Results

### Patients Characteristics

Data on the characteristics of the participants, including age, gender, duration of illness, diagnosis, and the test results used for diagnosis, are shown in Table 2. There were no significant differences between the two groups in any of these variables (sex: *p* = 1.00, age: *p* = 0.73, duration of illness: *p* = 0.116, diagnosis, *p* = 0.448).

**Table 2:** The clinical characteristics of the study cohort.

**Patient No/sex/age, y**	**Diagnosis**	**Duration of illness, mo**	**C-test Right**	**C-test Left**	**vHIT Right**	**vHIT Left**	**cVEMP**
**VRT group**							
1/m/40s	UVH	6	–	pathologic	–	pathologic	–
2/f/80s	BVH	12	pathologic	pathologic	–	–	pathologic
3/f/50s	UVH	30	–	pathologic	–	pathologic	–
4/m/30s	UVH	8	–	–	–	–	pathologic
5/f/50s	UVH	22	pathologic	–	–	–	–
6/m/70s	UVH	36	–	pathologic	–	pathologic	–
7/f/50s	UVH	30	pathologic	–	pathologic	–	pathologic
8/f/60s	UVH	15	–	pathologic	–	pathologic	pathologic
9/f/70s	UVH	31	–	–	–	–	pathologic
10/ m/70s	UVH	350	pathologic	–	pathologic	–	pathologic
11/ f/30s	UVH	18	–	–	–	–	pathologic
12/ f/40s	UVH	54	–	pathologic	–	pathologic	–
13/ m/50s	UVH	36	pathologic	–	–	–	–
14/ f/30s	BVH	30	pathologic	pathologic	pathologic	pathologic	–
15/ f/50s	UVH	30	–	–	–	–	pathologic
16/ f/40s	UVH	8	pathologic	–	–	–	–
17/ f/60s	BVH	30	pathologic	pathologic	pathologic	pathologic	–
18/ f/50s	UVH	11	–	–	–	pathologic	–
19/ f/70s	UVH	36	–	–	–	pathologic	pathologic
20/ f/60s	UVH	15	–	–	–	–	pathologic
21/ f/70s	UVH	80	–	–	–	–	pathologic
22/ f/70s	UVH	50	–	–	–	pathologic	pathologic
23/ f/70s	UVH	15	–	–	–	–	pathologic
24/ m/70s	UVH	35	pathologic	–	pathologic	–	pathologic
**Control group**							
1/m/70s	UVH	74	–	pathologic	–	pathologic	–
2/f/60s	UVH	30	pathologic	–	–	–	–
3/f/40s	UVH	40	–	–	–	–	pathologic
4/f/70s	UVH	36	pathologic	–	pathologic	–	–
5/m/60s	UVH	6	–	pathologic	–	–	–
6/f/50s	UVH	84	–	pathologic	–	pathologic	–
7/f/70s	UVH	23	–	–	–	–	pathologic
8/m/50s	UVH	70	–	–	–	pathologic	pathologic
9/f/70s	UVH	60	–	pathologic	–	pathologic	–
10/ f/70s	UVH	22	pathologic	–	pathologic	–	–
11/ f/60s	UVH	28	pathologic	–	pathologic	–	–
12/ f/70s	UVH	40	–	–	pathologic	–	–
13/ f/80s	UVH	35	–	pathologic	–	pathologic	–
14/ f/70s	UVH	11	–	–	pathologic	–	pathologic
15/ m/70s	UVH	120	pathologic	–	pathologic	–	–
16/ m/80s	UVH	48	–	–	–	pathologic	–
17/ f/50s	UVH	48	–	–	–	–	pathologic
18/ f/80s	BVH	48	pathologic	pathologic	pathologic	pathologic	–

*All patients had abnormal results in at least one of the three tests. A negative result implied that the test result was normal, while pathologic results indicated that it was abnormal*.

*m, male; f, female*.

*UVH, unilateral vestibular hypofunction; BVH, bilateral vestibular hypofunction*.

*C-test, caloric test; vHIT, video head impulse test; cVEMP, cervical vestibular evoked myogenic potentials*.

### Intra- and Inter-Group Comparisons of the Scores for Dizziness and Unsteadiness

**Figure 2:**
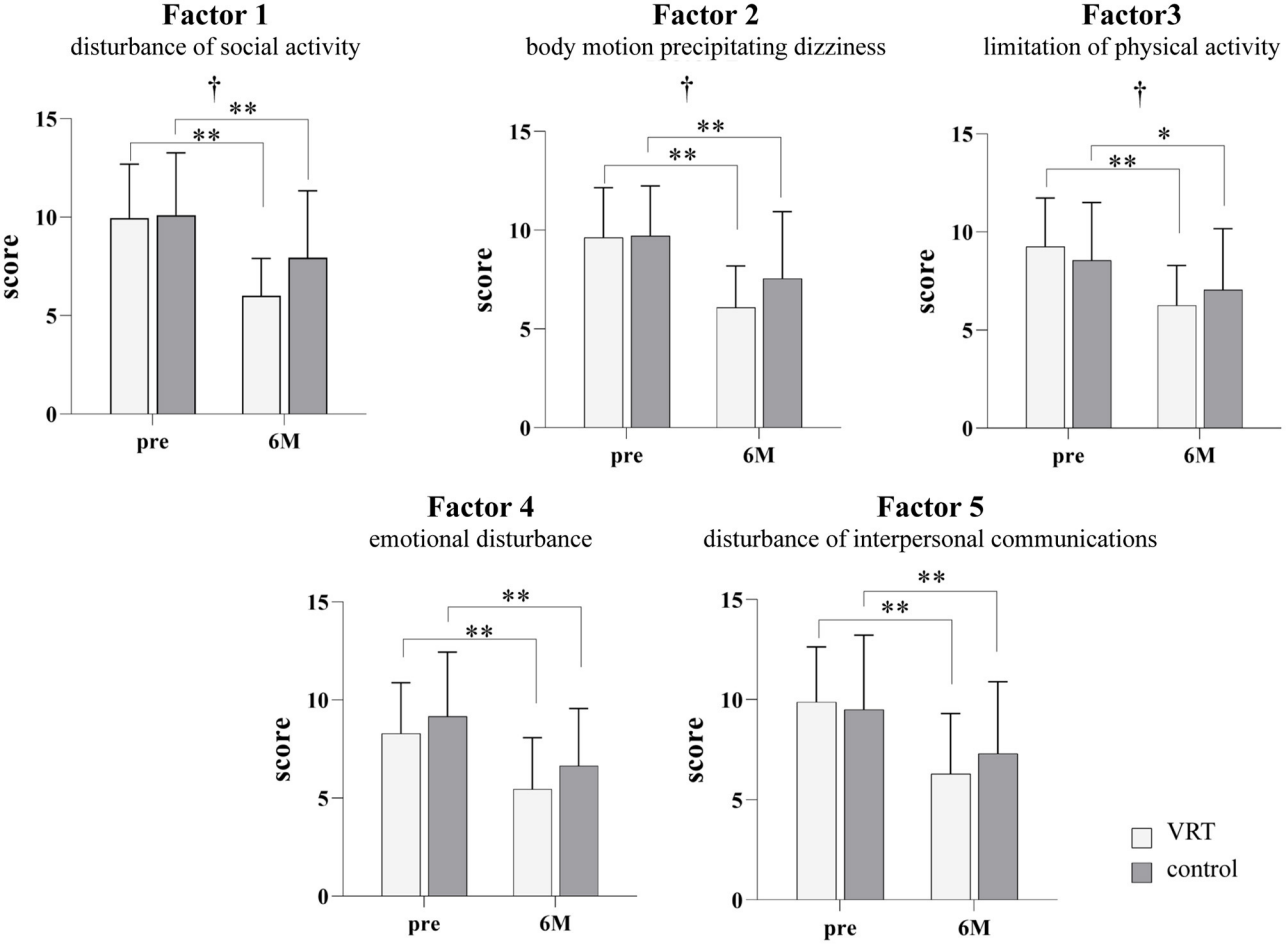
Dizziness and unsteadiness scores in the VRT and control groups before and after the six-month intervention. The white bars show the results for the VRT group, and the gray bars show the results for the control group. Significant interactions were found for factors 1 (F [1, 38] = 4.707; *p* = 0.0364), 2 (F [1, 40] = 4.497; *p* = 0.0402), and 3 (F [1, 38] = 4.330; *p* = 0.0442). There were no significant interactions for factors 4 (F [1, 40] = 0.179; *p* = 0.6745) and 5 (F [1,40] = 3.208; *p* = 0.0809). All factors showed a significant main effect of the time factor (factor 1: F [1, 38] = 55.18, *p* < 0.001; factor 2: F [1, 40] = 77.51, *p* < 0.001; factor 3: F [1, 38] = 38.97, *p* < 0.001; factor 4: F [1, 40] = 55.11, *p* < 0.001; factor 5: F [1, 40] = 55.52, *p* < 0.001). *Post-hoc* analysis demonstrated significant improvements in the scores for all factors after the completion of the 6-month intervention in both, VRT and control groups. (factor 1: VRT *p* < 0.001; control *p* = 0.002, factor 2: VRT *p* < 0.001; control *p* < 0.001, factor 3: VRT *p* < 0.001; control *p* = 0.018, factor 4: VRT *p* < 0.001; control *p* < 0.001, and factor 5: VRT *p* < 0.001; control *p* = 0.001) ^*^p < 0.05, ^**^p < 0.01, Bonferroni method. ^†^*p* < 0.05 interaction, two-way repeated measures ANOVA. VRT, vestibular rehabilitation therapy.

ANOVA (Figure 2) showed significant interaction between group and time factors and significantly higher improvements in the VRT than in the control group after the 6-month intervention, for the following factors: factors 1 (disturbance of social activity due to dizziness), 2 (body motion precipitating dizziness: head and sight), and 3 (limitation of physical activity: body movement). However, there were no significant interactions for factors 4 (emotional disturbance due to dizziness) and 5 (disturbance of interpersonal communications due to dizziness). The group factor showed no significant main effect on any factor in the questionnaire, and the time factor showed a significant decrease in scores of all factors after the intervention. *Post-hoc* analysis demonstrated significant improvements in scores of all the factors after completion of the 6-month-intervention, both in the VRT and control group.

### Correlation Between Improvement in Physical Activity and Subjective Dizziness

Table 3 shows the relationship between each average increase in physical activity and improvement in subjective dizziness. The change in physical activity was defined as the difference between the pre-intervention and after 2 and 6 months of intervention. In the VRT group, there was a significant negative correlation between the increase in SB and improvements in the scores for factors 1, 3, and 5 from before to 2 months after the initiation of the intervention; a significant positive correlation was noted between the increase in LPA and improvements in the scores of all the factors at the same time point. There were no significant correlations between increases in any of the physical activities and improvements in any of the five factors at the end of the intervention (month 6). In the control group, we did not observe any significant correlation between increases in any of the physical activities and improvement in the scores of any of the 5 factors at the 2- and 6-month time points.

**Table 3:** Correlation between improvement in physical activity and subjective dizziness.

	**Δ Factor 1** **disturbance of social activity**	**Δ Factor 2** **body motion precipitating dizziness**	**Δ Factor 3** **limitation of physical activity**	**Δ Factor 4** **emotional disturbance**	**Δ Factor 5** **disturbance of interpersonal communications**
**VRT**					
Increase in SB (2M)	**p = 0.007** **r = −0.539**	p = 0.098 r = −0.345	**p = 0.025** **r = −0.457**	p = 0.059 r = −0.391	**p = 0.006** **r = −0.541**
Increase in SB (6M)	p = 0.466 r = −0.156	p = 0.585 r = −0.117	p = 0.131 r = −0.318	p = 0.721 r = −0.077	p = 0.270 r = −0.234
Increase in LPA (2M)	**p < 0.001** **r = 0.689**	**p = 0.043** **r = 0.417**	**p = 0.016** **r = 0.486**	**p = 0.011** **r = 0.511**	**p = 0.001** **r = 0.640**
Increase in LPA (6M)	p = 0.440 r = 0.165	p = 0.791 r = 0.057	p = 0.215 r = 0.263	p = 0.963 r = −0.010	p = 0.468 r = 0.156
Increase in MVPA (2M)	p = 0.550 r = 0.128	p = 0.916 r = −0.023	p = 0.758 r = −0.066	p = 0.440 r = −0.165	p = 0.605 r = 0.111
Increase in MVPA (6M)	p = 0.108 r = 0.337	p = 0.427 r = 0.170	p = 0.706 r = 0.081	p = 0.749 r = 0.069	p = 0.189 r = 0.278
**Control**					
Increase in SB (2M)	p = 0.230 r = −0.298	p = 0.359 r = −0.230	p = 0.702 r = −0.097	p = 0.935 r = −0.021	p = 0.637 r = −0.119
Increase in SB (6M)	p = 0.121 r = −0.379	p = 0.287 r = −0.265	p = 0.312 r = −0.252	p = 0.607 r = −0.130	p = 0.284 r = −0.267
Increase in LPA (2M)	p = 0.306 r = 0.255	p = 0.499 r = 0.170	p = 0.303 r = 0.257	p = 0.976 r = 0.008	p = 0.603 r = 0.131
Increase in LPA (6M)	p = 0.080 r = 0.423	p = 0.285 r = 0.267	p = 0.112 r = 0.388	p = 0.266 r = 0.277	p = 0.129 r = 0.372
Increase in MVPA (2M)	p = 0.489 r = 0.255	p = 0.499 r = 0.170	p = 0.303 r = 0.257	p = 0.976 r = 0.008	p = 0.603 r = 0.131
In MVPA(6M)	p = 0.080 r = 0.423	p = 0.285 r = 0.267	p = 0.112 r = 0.388	p = 0.266 r = 0.277	p = 0.129 r = 0.372

*Numbers with significant correlations are shown in bold characters*.

*SB, sedentary behavior; LPA, light-intensity physical activity; MVPA, moderate to vigorous-intensity physical activity*.

### Comparison of the Percentage Increase in Physical Activity Between Groups

**Figure 3:**
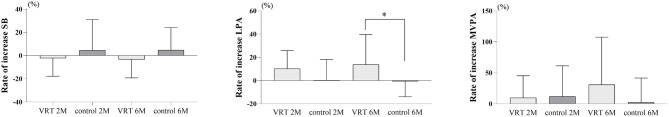
Comparison of the percentage increase in physical activity between the groups. The VRT group showed a significantly higher LPA than did the control group after 6 months of intervention (VRT: 13.9 ± 25.2%; control: −0.7 ± 12.8%; mean difference [MD]: 14.6 ± 6.7%; 95% confidence interval [CI]: 1.1–28.0%; *p* = 0.0347). There was no significant difference between the two groups in the rate of increase in SB in month 2 (VRT: −2.4 ± 15.1%; control: 4.7 ± 25.6%; MD: −7.1 ± 6.5%; 95% CI: −20.2–6.0%; *p* = 0.2799) or month 6 of the intervention (VRT: −3.4 ± 15.5%; control: 4.8 ± 18.6%; MD: −8.3 ± 5.4%; 95% CI: −19.1–2.6%; *p* = 0.1326). No significant difference was also observed between the groups for LPA (VRT: 10.4 ± 15.1%; control: 0.3 ± 17.4%; MD: 10.1 ± 5.1%; 95% CI: −0.3–20.5%; *p* = 0.0559) or MVPA (VRT: 17.7 ± 48.5%; control: 12.1 ± 47.8%; MD: 5.5 ± 15.3%; 95% CI: −25.6–36.6%, *p* = 0.7218) after 2 months of intervention. The MVPA was also not significantly different between the groups at the end of the intervention (VRT: 31.1 ± 74.8%; control: 2.0 ± 38.3%; MD: 29.1 ± 19.8%; 95% CI: −10.8–69.0%; *p* = 0.1485). ^*^p < 0.05, unpaired *t-test*. SB, sedentary behavior; LPA, light-intensity physical activity; MVPA, moderate-to-vigorous-intensity physical activity.

Figure 3 shows the difference in the ratio of change in physical activity between the VRT and control groups. The ratio of change in physical activity was the quotient of physical activity at pre-intervention by that after 2 and 6 months of intervention. The unpaired *t-test* revealed that the VRT group showed a significantly higher increase in LPA than the control group at the end of the intervention. There was no significant difference between the two groups in terms of the rate of increase in SB at month 2 or 6 of the intervention. No significant difference was observed between the groups in terms of LPA or MVPA after 2 months of intervention. The MVPA was not significantly different between the groups at the end of the intervention.

All data are available online in the data storage (http://dx.doi.org/10.17632/v8d9hfk84b.1).

## Discussion

The major findings of the present study were as follows: (1) supervised VRT by PTs was considerably effective in improving subjective dizziness; (2) in supervised VRT, an increase in the LPA at an early stage of the intervention was directly associated with a better outcome, with higher increases in LPA resulting in more reduction in subjective dizziness at the end of the intervention; and (3) the supervised VRT group also had significantly better final ratios of LPA than the control group.

In the present study, we found that improvement in subjective dizziness in the VRT group was higher for three factors: disturbance of social activity due to dizziness, body motion precipitating dizziness (head and sight), and limitation of physical activity (body movement). This indicates that supervised VRT can effectively promote vestibular compensation. Both groups exercised, but the VRT group performed daily voluntary exercises aimed at improving the dizziness; this may explain the difference in this effect. To promote vestibular compensation, movements that are difficult to perform and are likely to cause dizziness must be completed; frequent PT-guided interventions are crucial in reducing fear while correcting the mistakes in exercise methods. There was no significant interaction between the factors of emotional disturbance due to dizziness and disturbance in interpersonal communication due to dizziness; this suggests that supervised VRT may be involved in improving motor abilities rather than mental effects on dizziness. A number of previous studies have also reported improvements in subjective dizziness and objective measures such as postural control ability after individualized VRT administered by PTs (9–13). However, the control group, which received only lifestyle guidance, showed improvement in all factors of subjective dizziness; however, the effects varied. The commonality between the two interventions was that the diagnosis was clarified through inpatient testing, and the interventions provided explanations and lifestyle guidance to eliminate the fear of vertigo. Yardley reported that having completed the medical investigation, it is important to present the diagnostic conclusions in a positive light. Educating patients about the role of activity in recovery also provides the first step toward encouraging active self-management of symptoms. These patients can be encouraged to resume activity simply by explaining that movement-provoked symptoms are an inevitable part of the recovery process and are not warning signals of possible serious damage and illness (3). The improvement in dizziness symptoms in both groups in the present study may have been influenced by a well-defined explanation. While most previous studies have reported treatment effects over a short period of time, such as 4 weeks, the present study examined the effects of the intervention over a longer period of 6 months. Jung et al. (28) described an improvement in subjective dizziness in the non-VRT group at 3 months of follow-up. It can be argued that patients with chronic dizziness can improve in the long-term with proper diagnosis and lifestyle guidance alone; however, VRT under the supervision of a physical therapist can enhance this improvement. It is possible that the effect of VRT under supervision is influenced by both, the promotion of vestibular compensation by targeted exercises and the increase in activity by lifestyle guidance.

The relationship between increased physical activity and improvement in subjective dizziness at the end of the intervention was significantly correlated with a decrease in SB, an increase in LPA, and an improvement in subjective dizziness at 2 months after the initiation of VRT. This relationship was not found in the control group. The improvement in subjective dizziness with VRT was influenced by increased activity early after the initiation of the intervention. Patients with chronic vertigo have been reported to have higher rates of SB and lower rates of LPA than healthy individuals (4), and it is possible that the patients in this study may have suffered from low activity as well as primary vertigo symptoms. Patients with dizziness in the chronic phase have been found to have an exacerbation of dizziness secondary to decreased activity due to a vicious cycle of dizziness, in addition to dizziness caused by peripheral vestibular disorders; intervention for these two factors may enhance the effectiveness in improving dizziness. There was no relationship between the amount of physical activity and improvement in dizziness in the control group, indicating that exercises that promote vestibular compensation and increase physical activity may have a major role in improving subjective dizziness. We observed that patients with chronic dizziness should increase their physical activity for vestibular compensation, and VRT under PT monitoring may enhance the effect. There was no correlation between the increase in physical activity and improvement in subjective dizziness after 6 months, suggesting that the 2-month change observed in the present study may be related to the improvement in dizziness due to an early increase in activity, rather than an increase in activity owing to an improvement in dizziness.

Finally, the rate of improvement in LPA after 6 months was significantly higher in the VRT group than in the control group. It was considered that frequent PT-guided VRT may increase the rate of LPA in the daily life of patients with chronic dizziness. It is also possible that the increase in activity exacerbated the symptoms of vertigo temporarily; this may have made to adherence to lifestyle guidance more difficult in the control group than in those receiving supervised VRT. Using a 3-month booklet-based VRT in a voluntary practice setting, previous studies have shown that high adherence was observed in only 37.5% of patients; this was attributed to worsening of symptoms following the initiation of voluntary exercises (29). Supervised VRT by a PT may be effectively continued until the symptoms of dizziness improve, even if the dizziness worsens temporarily. A multicenter, randomized, controlled trial on a physical activity promotion program by PTs for community-dwelling stroke patients reported no change in physical activity after 18 months to 2 years of intervention (30, 31). There was a significant difference between the participants, and patients with chronic dizziness may have shown decreased activity owing to the vicious cycle of dizziness-anxiety caused by physical activity. Patients with dizziness show discrepancies in movement ability and activity, and we believe that correcting this discrepancy may encourage an increase in physical activity. In VRT, interventions in physical activities of daily living are important not only in terms of vestibular compensation, but also in terms of improving the quality of life.

This study has some limitations. A major limitation is the design of the controlled intervention; the fact that patients in the VRT group had weekly contact with a therapist, while those in the control group attended only one counseling session every 2 months may have led to biased results. The improvement in subjective dizziness, which was the outcome of this study, may be affected by the frequency of contact; in addition, the effect of VRT in this study may have been influenced by the frequency of intervention. However, since the therapist and the evaluator were different, the bias due to the frequency of intervention was considered to be less. Since this was a randomized, controlled study, we were able to clearly determine the difference in treatment effects between supervised VRT for patients with peripheral vestibular disorders and the standard practice in Japan, which is to provide lifestyle guidance to these patients. In addition, the effect of the intervention was only evaluated in terms of subjective dizziness. Since the process of vestibular compensation involves improvements in balance and fixation, objective measures need to be investigated to examine the progression of vestibular compensation in detail. However, the VRT questionnaire used in this study assessed subjective dizziness in detail by dividing the factors into five groups. There were no differences in factor 4 and emotional disturbance, and there was an interaction between factors 2 and 3; this reflected dynamic vestibular compensation, and indicated that the VRT used in this study had an effect on vestibular compensation. Future research should examine the differences in the effectiveness of VRT depending on the type of disease, so that appropriate treatment options can be selected on the basis of individual characteristics of each patient.

## Conclusion

In patients with chronic dizziness, frequent (weekly) and on-going (for 6 months) supervised VRT supported by PTs was effective in improving subjective dizziness. The effectiveness of supervised VRT could be enhanced by encouraging a high level of LPA at the early stage of intervention; this should be maintained thereafter. Therefore, VRT sessions under the supervision of PTs could promote an increase in physical activity and improve the quality of life in patients with chronic dizziness.

## Data Availability Statement

The raw data supporting the conclusions of this article will be made available by the authors, without undue reservation.

## Ethics Statement

The studies involving human participants were reviewed and approved by Ethics Committee of Nara Medical University Hospital. The patients/participants provided their written informed consent to participate in this study.

## Author Contributions

TS: study design, data interpretation, and writing. TI: data collection and analysis. YW: patient allocation. TY and TK: data interpretation. All authors contributed to the article and approved the submitted version.

## Conflict of Interest

The authors declare that the research was conducted in the absence of any commercial or financial relationships that could be construed as a potential conflict of interest.
